# The treatment effect of posterior lumbar fusion surgery on patients suffering from lumbar disc herniation concurrent with peroneal nerve paralysis

**DOI:** 10.3389/fsurg.2022.1063528

**Published:** 2023-01-06

**Authors:** Shangju Gao, Zhaohui Li, Xiangyu Li, Samuel Rudd, Haoming Wang, Ze Gao, Wenyuan Ding, Sidong Yang

**Affiliations:** ^1^ Department of Spine Surgery, The Third Hospital of Hebei Medical University, Shijiazhuang, China; ^2^ Department of Orthopedic Surgery, Hebei General Hospital, Shijiazhuang, China; ^3^ School of Chemical Engineering, The University of Queensland, QLD, Brisbane, Australia

**Keywords:** lumbar disc herniation, peroneal nerve paralysis, lumbar fusion, lower back pain, risk factor, foot drop

## Abstract

**Purpose:**

The purpose of this study is to investigate the clinical effect of posterior lumbar fusion surgery on patients who suffer from lumbar disc herniation concurrent with peroneal nerve paralysis.

**Methods:**

The patients suffering from peroneal nerve paralysis and undergoing posterior lumbar fusion surgery between January 2012 and December 2019 were retrospectively reviewed. The data of the identified patients were then collected and processed. All patients were followed up post-operatively after discharge from the hospital. The data was analyzed in terms of Oswestry disability index (ODI), visual analogue scale (VAS) score, and relative lower-limb muscle strength.

**Results:**

A total of 87 patients (52 males and 35 females) aged 54 ± 11 years met the inclusion criteria for this study. These patients stayed in hospital for 16 ± 6 days and were followed up for 81 ± 24 months. Data analysis showed that muscle strength of the tibialis anterior and extensor digitorum significantly recovered at the last follow-up with a grade of 3 (median), compared to grade 0 at admission (*p* < 0.001). Furthermore, the median VAS score decreased to 1 at the last follow-up from 6 at admission (*p* < 0.001), and the ODI greatly improved with 10% (median) at the last follow-up, while it was 58% at admission (*p* < 0.001). The ODI improvement rate was 60% on average at the last follow-up. Multivariate regression analysis regarding the ODI and muscle strength improvement rates showed that advanced age was a risk factor for postoperative recovery.

**Conclusions:**

Most of the patients suffering from lumbar disc herniation concurrent with peroneal nerve paralysis can improve after undergoing posterior lumbar fusion surgery, but few can reach full recovery. Advanced age might be a risk factor that affects the prognosis of these patients after surgery.

## Introduction

In clinical scenarios, lumbar disc herniation (LDH) has a high incidence, most cases being caused by intervertebral disc degeneration (IVDD) (1–3). LDH can lead to lower back pain (LBP), radicular pain and numbness of lower limbs, and even peroneal nerve paralysis (4–7). The clinical symptoms caused by peroneal nerve paralysis include foot and toe (hallux) drop, which results from weakness of ankle (tibialis anterior) and toe dorsiflexion (extensor digitorum) (8). Foot drop has been reported to have an incidence of 0.6%–7.7% in lumbar IVDD diseases, most of which are LDH cases (9).

For these LDH patients with peroneal nerve paralysis, lumbar surgeries are usually performed to remove the herniated nucleus pulposus (the disc), decompress the nerve root and enlarge spinal canal. Nowadays, posterior lumbar surgery, with or without interbody fusion, is widely used to treat LDH, particularly for those cases concurrent with peroneal nerve paralysis (10). However, some previous studies indicated that patients after lumbar surgery might experience prolonged LBP which significantly lowers their quality of life (11–14). Although there have been some studies on LDH-induced peroneal nerve paralysis so far, it is still difficult to make definitive conclusions based on these studies considering the variety of surgical procedures used (4).

Thus, the purpose of this study is to investigate the clinical effect of posterior lumbar fusion surgery on patients who suffered from LDH concurrent with peroneal nerve paralysis.

## Patients and methods

### Ethics

This retrospective study has been approved by Medical Ethics Council of the Third Hospital of Hebei Medical University (approval no. K2022-127-1). All informed consent was obtained from the patients (or their lawful guardians).

### Patients

**Figure 1:**
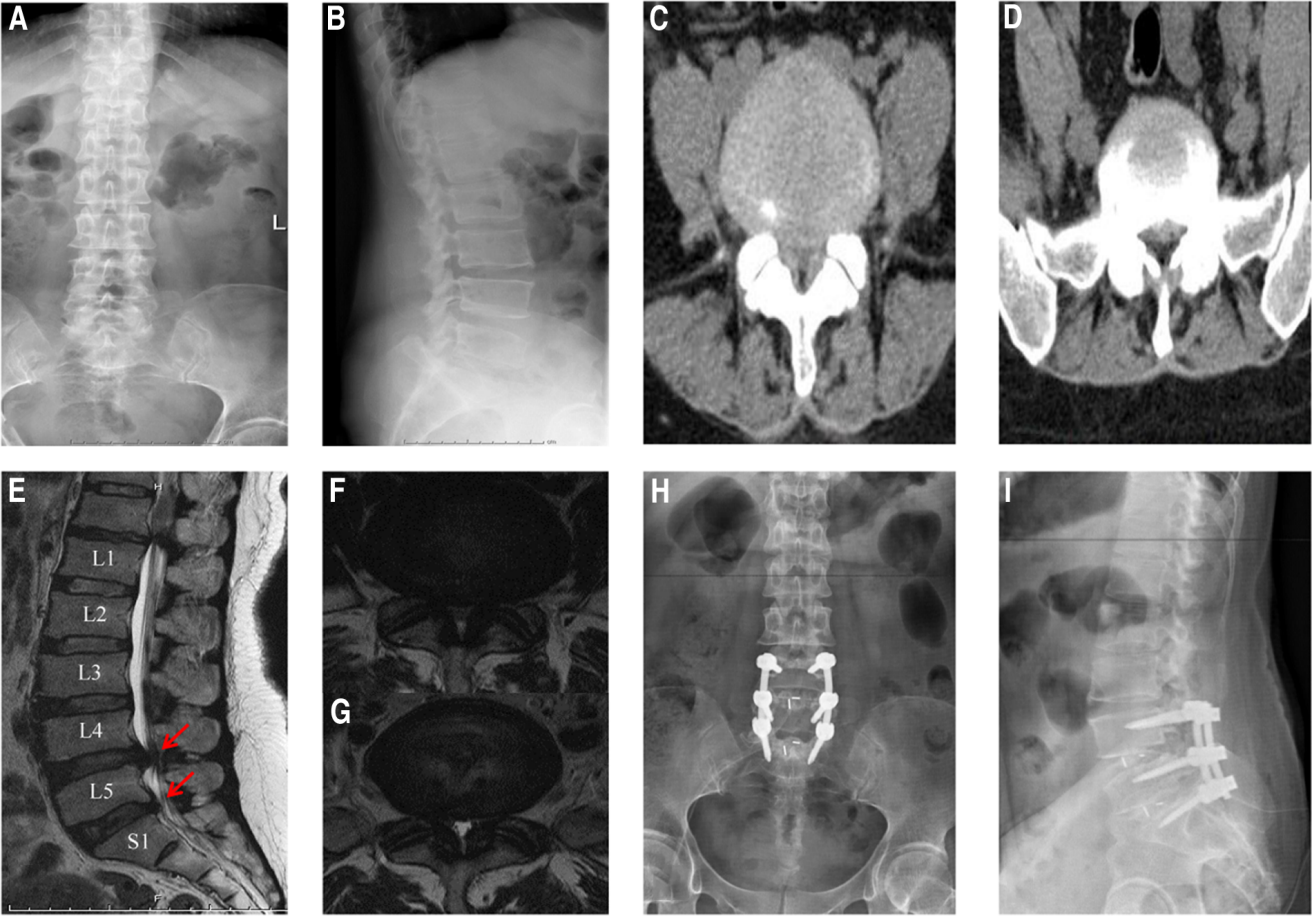
A representative case of posterior fusion surgery. (**A,B**) Preoperative x-ray radiographs; (**C,D**) preoperative CT scans of L4-5 and L5-S1, respectively; (**E**) preoperative MRI scan (sagittal plane); (**F,G**) preoperative MRI scans of L4-5 and L5-S1, respectively (axial plane); (**H,I**) postoperative x-ray radiographs. The arrows indicate the herniation of intervertebral disc.

The patients who were diagnosed with LDH and peroneal nerve paralysis between January 2012 and December 2019 were retrospectively reviewed. All participants in this study underwent posterior lumbar fusion surgery (Figure 1) as previously reported (15). The data of the identified patients were then collected and processed. All patients were followed up after discharge from the hospital.

### Assessment

The data collected in this study included baseline data and functional parameters. Baseline data consisted of age, gender, hospital stay, blood loss, operation time, follow-up time, and patient satisfaction. Functional parameters included Oswestry disability index (ODI), visual analogue scale (VAS) score, and lower-limb muscle strength. The lower-limb muscle strength was scored and analyzed by assessing muscle strength of the tibialis anterior and extensor digitorum, using the muscle scale established by the Medical Research Council (16). Foot drop and toe drop are defined as muscle strength below or equal to grade 3 (out of 5) (17). Additionally, the patients' satisfaction was collected and graded to three levels; dissatisfied, satisfied, and very satisfied.

ODI improvement rate was calculated using the equation (18, 19):

$$\left(1-\frac{Postoperative\;ODI}{Preoperative\;ODI}\right)\times 100\%$$

Muscle strength improvement rate was calculated using the equation:

$$\frac{Postoperative\;strength-Preoperative\;strength\;}{5-Preoperative\;\mathrm{strength}}\times 100\%$$

To identify the risk factors that affect postoperative recovery, multivariate regression analyses were performed in terms of the ODI improvement rate and muscle strength improvement rate, respectively.

### Statistics

SPSS for Windows 18.0 (SPSS Inc, US) was used for statistical analysis in this study. The data of ODI, VAS score and muscle strength is presented as median and interquartile range (IQR). The other data is presented as mean ± standard deviation (SD). Mann–Whitney *U* tests were used to analyze ODI, VAS score and muscle strength between pre-operation and post-operation. In addition, multivariate regression analyses (Enter method)were performed to identify the risk factors that would affect postoperative recovery in terms of the ODI improvement rate and muscle strength improvement rate, respectively. *p* values less than 0.05 were regarded as significant.

## Results

### Baseline data

After screening and review of the patients who had undergone lumbar fusion surgeries between January 2012 and December 2019, a total of 87 patients were identified and included in this study. As shown in Table 1, there are 52 males and 35 females. The age of these participants was 54 ± 11 years. The hospital stay was 16 ± 6 days, and blood loss was 752 ml on average. Operation time was 196 min on average, and the follow-up time for these patients was 81 ± 24 months. Median time to surgery was 1 month. Among all participants, most were very satisfied or satisfied about their treatment effects (78 in 87) and only 9 patients were dissatisfied.

**Table 1:** Baseline clinical data of patients (*n* = 87).

Parameters	Mean	SD
Age (years)	54	11
Gender (male/female)	52/35	
Hospital stay (day)	16	6
Blood loss (ml)	752	471
Operation time (min)	196	69
Time to surgery (month)	1 (3)*	
Follow-up time (month)	81	24
Satisfaction (very satisfied/satisfied/dissatisfied)	49/29/9	

SD, standard deviation.

* Median (interquartile range).

### Interbody fusions

**Figure 2:**
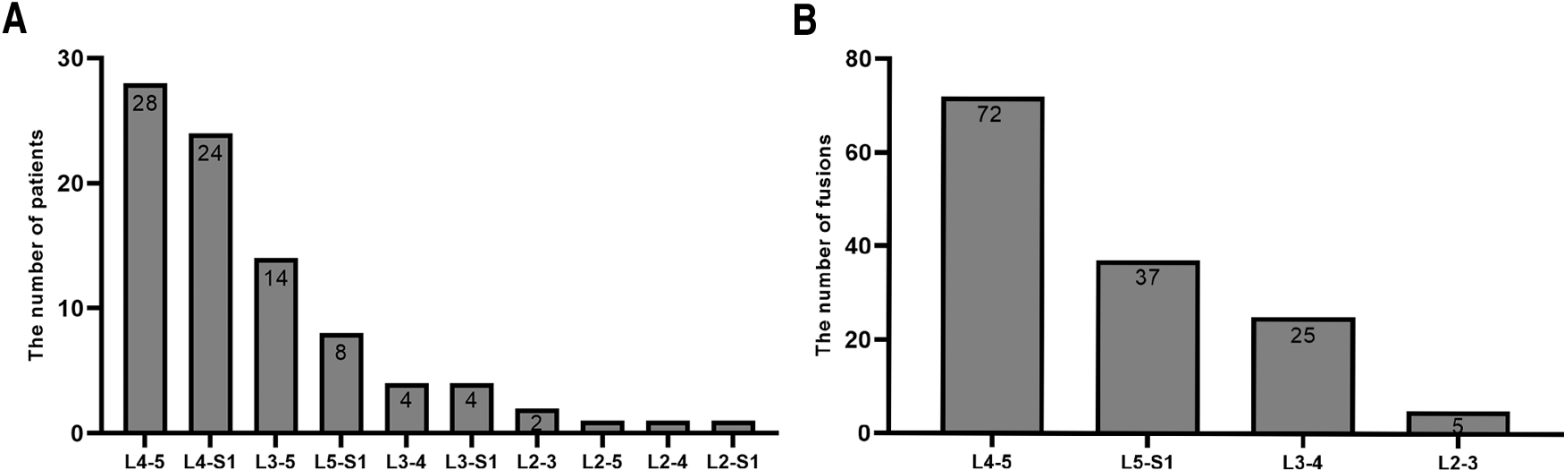
Lumbar fusions performed to the patients. (**A**) The number of patients in terms of fusions; (**B**) the number of lumbar fusions in terms of every fused segment.

As shown in Figure 2A, all participants underwent posterior lumbar interbody fusion surgeries. Among all 87 patients, 28 underwent L4-5 fusions, 24 underwent L4-S1 fusions, 14 underwent L3-5 fusions, and 21 underwent other segments’ fusions. As shown in Figure 2B, there were a total of 139 interbody fusions performed across all 87 patients. The majority of these fusions were L4-5 fusions and L5-S1 fusions, while the fusions of L3-4 and L2-3 were less common.

### Muscle strength and improvement rate

**Figure 3:**
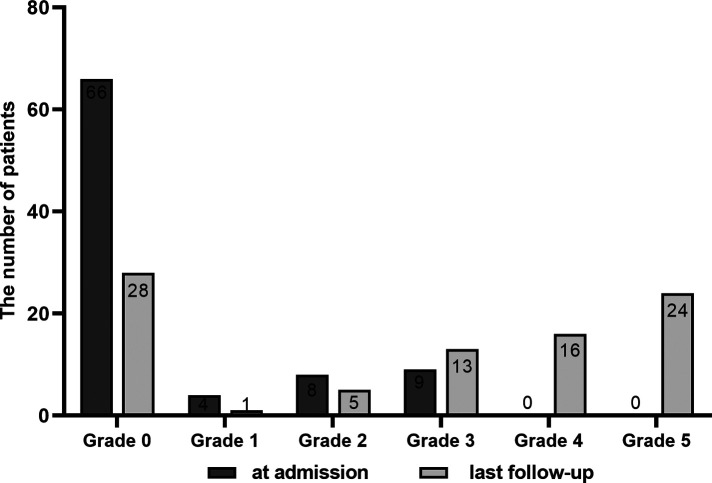
Comparison of muscle strength between pre-surgery (at admission) and last follow-up after surgery.

As shown in Figure 3, 66 of 87 patients were at grade 0 muscle strength of the tibialis anterior and extensor digitorum at admission, and 40 of the 87 patients improved to grades 4 or 5 at the last follow-up. Among 87 patients, 56 (64.4%)improved their muscle strength. As shown in Table 2, overall, the median preoperative muscle strength of the tibialis anterior and extensor digitorum was grade 0 at admission and grade 3 at the last post-operative follow-up. Compared to the preoperative gradings, the patients' muscle strength significantly recovered after surgery (*p* < 0.001). Muscle strength improvement rate was 50% on average at the last follow-up.

**Table 2:** Comparisons regarding muscle strength, VAS, and ODI (median and IQR).

	Muscle strength	VAS	ODI
At admission	0 (0)	6 (5)	58% (60%)
Last follow-up	3 (5)	1 (2)	10% (26%)
*P* valve	***<0.001***	***<0.001***	***<0.001***

VAS, visual analogue scale; ODI, Oswestry disability index; IQR, interquartile range.

### VAS score

As shown in Table 2, the median VAS score of the patients was 6 at admission. By contrast, the median VAS score decreased to 1 at the last post-operative follow-up. Statistical analysis showed that the patients' post-operative VAS score significantly decreased compared with pre-operation (*p* < 0.001).

### ODI and ODI improvement rate

As shown in Table 2, the patients' median ODI was 58% at admission, and decreased to 10% at the last post-operative follow-up (*p* < 0.001). The ODI improvement rate was 60% on average at the last follow-up.

### Multivariate regression analysis

In this study, multivariate regression analysis was performed to identify risk factors that may influence the recovery of the patients after surgery. In the regression analyses, ODI improvement rate and muscle strength improvement rate were used as the dependent variables, respectively. It showed that advanced age was a risk factor that might affect the final ODI improvement rate and muscle strength improvement rate (both *p* < 0.05), as shown in Tables 3, 4.

**Table 3:** Multivariate regression analysis regarding ODI improvement rate.

	Unstandardized		Standardized		
Parameters	B	SE	Beta	*t*	*p*
Gender	−5.114	11.745	−0.044	−0.435	0.665
Age	−1.493	0.588	−0.275	−2.540	***0*** *.* ***013***
Hospital stay	−0.301	1.017	−0.030	−0.296	0.768
Number of intervertebral fusions	−16.557	12.021	−0.189	−1.377	0.172
Blood loss	0.020	0.018	0.163	1.099	0.275
Operation time	0.036	0.115	0.044	0.318	0.752
Follow-up time	−0.488	0.262	−0.199	−1.867	0.066
Time to surgery	0.177	0.143	0.128	1.242	0.218
Muscle strength at admission	1.592	5.425	0.029	0.293	0.770
VAS at admission	−1.297	2.709	−0.072	−0.479	0.634
ODI at admission	0.898	0.271	0.510	3.310	***0***.***001***

VAS, visual analogue scale; ODI, Oswestry disability index.

**Table 4:** Multivariate regression analysis regarding muscle strength improvement rate.

Parameters	Unstandardized		Standardized Beta	*t*	*p*
	B	SE			
Gender	3.289	9.502	0.039	0.346	0.730
Age	−1.015	0.477	−0.258	−2.127	***0***.***037***
Hospital stay	0.336	0.832	0.046	0.403	0.688
Number of intervertebral fusions	3.000	9.793	0.047	0.306	0.760
Blood loss	−0.003	0.015	−0.031	−0.187	0.852
Operation time	−0.084	0.093	−0.139	−0.902	0.370
Follow-up time	−0.150	0.211	−0.085	−0.713	0.478
Muscle strength at admission	8.068	4.294	0.206	1.879	0.064
VAS at admission	−0.936	2.190	−0.072	−0.427	0.670
ODI at admission	0.272	0.217	0.214	1.254	0.214
Time to surgery	−0.217	0.554	−0.045	−0.392	0.696

VAS, visual analogue scale; ODI, Oswestry disability index.

## Discussion

Clinically, pathological changes in neural structures that influence the dorsiflexion of ankle would cause peroneal nerve paralysis, such as nerve compression, trauma, infection, tumor, and external oppression (5). LDH is one of the common diseases that lead to peroneal nerve paralysis (7, 8). Clinical symptoms caused by peroneal nerve paralysis usually include foot and toe (hallux) drop, resulting from weakness of the ankle (tibialis anterior) and toe (extensor digitorum) dorsiflexion. As previously reported, L4-5 disc herniation is the most common LDH that causes peroneal nerve paralysis, and L5-S1 disc herniation contributes 25% of cases with peroneal nerve paralysis (4, 5). In clinical settings, these LDH patients need to undergo lumbar spine surgeries regardless of interbody fusions. In the current study, we retrospectively collected 87 patients who underwent posterior lumbar fusion surgeries to treat LDH with peroneal nerve paralysis. The purpose of this study is to investigate the clinical effect of posterior lumbar fusion surgery on patients who suffered from LDH concurrent with peroneal nerve paralysis.

After being reviewed and identified, a total of 87 participants are finally enrolled in our study. Compared with pre-surgery, the functional parameters have significantly improved after surgery in terms of VAS score, lower-limb muscle strength, and ODI score. Moreover, most patients are very satisfied or satisfied about their treatment effects after fusion surgery. Our findings are in line with some existing reports indicating postoperative pain relief and functional recovery after the patients underwent lumbar surgeries to remove protruded disc and decompress nerve roots (4, 5, 9).

In our study, 56 out of 87 patients had improvements of muscle strength. Overall, the median muscle strength of the tibialis anterior and extensor digitorum improved to a grade of 3 at the last follow-up from grade 0 at admission. Among all 87 patients, there are 40 (46%) patients with muscle strength of grades 4 or 5 at last follow-up. Liu et al. (17) reported 135 patients who suffered from lumbar degenerative diseases with foot drop, and all these patients underwent posterior lumbar interbody fusion surgery with pedicle screw instrumentation. Their study shows 83.7% cases improved in muscle strength; however, only 15.6% patients improved to grades ≧4. By contrast, our study has shown a higher improvement rate (46%, grades ≧4) in muscle strength for the patients who had undergone lumbar fusion surgeries because of LDH and peroneal nerve paralysis. In addition, a multivariate regression analysis of our study shows that advanced age is a risk factor that may affect the final recovery of postoperative patients. This finding is consistent with Liu et al. (17) who reported that younger patients more often have a better surgical outcome.

To date, some studies have proposed a few risk factors and prognosis factors that influence post-operative recovery, but there is no consensus in this aspect. Shorter duration of peroneal nerve paralysis (17, 20), better pre-operative muscle strength (17, 20–22), shorter time to surgery (22), and younger age (17, 23) have been reported to indicate better recovery outcomes for patients who undergo lumbar surgeries due to lumbar spine diseases with peroneal nerve paralysis. However, it has been reported that there are no significant associations between postoperative recovery and the factors including age, diagnosis (LDH or spinal stenosis), duration of symptoms, and preoperative muscle strength (24).

Previous studies (25–27) have indicated that lower-limb exercise can effectively facilitate post-operative pain relief and promote functional recovery of patients undergoing spinal surgery. However, it still remains controversial regarding whether postoperative lower-limb exercise can really accelerate postoperative recovery. Some studies reported that postoperative lower-limb exercise can increase pain relief, functional improvement and patient satisfaction (25–30), while some others did not show positive effects of postoperative lower-limb exercise on final recovery (31–33).

There are some limitations restricting the data interpretation of this study. To start with, this is a single-center retrospective study, and as such the participants lack extensive representation which may affect the accuracy of the data. In addition, the patient sample size is not large, as only 87 participants were included in this study. The results and conclusions would be more robust if the patient sample size was larger. Hence, a larger and therefore more comprehensive study is needed to address all the issues above. The preferred study design would be multi-center, prospective, blinded and randomly controlled, with a larger sample size.

## Conclusions

Most of patients suffering from lumbar disc herniation concurrent with peroneal nerve paralysis can improve after undergoing posterior lumbar fusion surgery, but few can reach full recovery. Advanced age might be a risk factor that affects the prognosis of these patients after surgery.

## Data Availability

The raw data supporting the conclusions of this article is available from the corresponding authors upon request.
